# A Rare Case of West Nile Virus-Associated Cardiomyopathy

**DOI:** 10.7759/cureus.28473

**Published:** 2022-08-27

**Authors:** Ashley R Gao, Laura Nichols, Devendranath Mannuru

**Affiliations:** 1 Internal Medicine, University of North Dakota School of Medicine and Health Sciences, Fargo, USA; 2 Department of Internal Medicine, Sanford Health, Fargo, USA

**Keywords:** west nile virus infection, west nile fever, echocardiography, west nile virus myocarditis, west nile virus cardiomyopathy

## Abstract

A 68-year-old man presented in late summer 2021 with fever, myalgias, generalized weakness, dizziness, and headache. Past medical history included rheumatoid arthritis treated with infliximab, congestive heart failure with preserved ejection fraction, and recent travel to Alaska. He was febrile, tachycardic, and tachypneic on admission. Physical exam and admission labs were overall unremarkable. On day 4, he complained of shortness of breath and central chest discomfort. Troponin was mildly elevated, electrocardiogram was unremarkable, and echocardiogram showed new global wall motion abnormalities and ejection fraction of 40%, which was 55% months prior. Serum West Nile IgM antibodies resulted positive near the end of hospitalization. Testing for SARS-CoV-2, influenza as well as multiple other viral, bacterial, and fungal organisms was negative. Overall, the patient recovered clinically including improvement in ejection fraction on echocardiogram with conservative management. West Nile virus (WNV) is associated with a myriad of symptoms and complications, most notably, neuroinvasive disease. However, cardiomyopathy secondary to WNV as illustrated in this case has been infrequently described. Clinicians should be aware of this potential rare complication in patients with WNV to improve rapid detection and treatment of myositis, associated cardiomyopathy, and related complications.

## Introduction

West Nile virus (WNV) is a mosquito-borne member of the Flaviviridae family endemic to North America, Europe, Africa, the Middle East, South Asia, and Australia [1]. First isolated in Uganda in 1937, a series of outbreaks in the Middle East and Europe in the 1990s eventually introduced the virus to the Western Hemisphere in 1999. WNV is sustained in nature in a cycle between birds and mosquitoes. Humans and other affected mammals are dead-end hosts. While the main mode of transmission to humans is through a mosquito bite, other modes such as intrauterine transmission between infected mothers and fetuses, through breastfeeding, blood transfusions, and organ transplants have also been previously documented [2].

While 80% of infected individuals are asymptomatic, patients who have symptoms primarily experience a mild flu-like illness, termed West Nile Fever (WNF), after an incubation period of 2-15 days. A very small subset, about 1%, of symptomatic individuals progress to neuroinvasive disease; at risk are those with underlying risk factors such as older age, hematological malignancies, and immunosuppression [1-3]. Myocarditis is a very infrequent complication that is not well-documented; subsequent cardiomyopathy is even rarer.

## Case presentation

A 68-year-old immunocompromised Caucasian man presented in late summer 2021 with fever, myalgias, generalized weakness, dizziness, and headache for 3 days. Past medical history included rheumatoid arthritis, for which he received infliximab infusions every 6 weeks, and congestive heart failure with preserved ejection fraction. He also reported recent travel to Alaska and possible tick exposure. On admission, he was febrile to 101.6°F, tachycardic, and tachypneic. Physical examination was otherwise unremarkable. Labs were notable for a white blood cell count of 2.4 K/uL (reference range 4.0-11.0 K/uL) and aspartate aminotransferase of 69 U/L (reference range 0-35 U/L). He was initially started on broad-spectrum antibiotics with vancomycin and cefepime due to concern for sepsis secondary to a bacterial source. COVID-19, influenza virus, ehrlichiosis, anaplasmosis, and Lyme disease were also a part of the initial workup, and all returned negative (Table 1). Blood and urine cultures were also negative. He continued to have fevers and infectious disease was consulted. Vancomycin was subsequently discontinued, and the patient was initiated on doxycycline for coverage of possible tick-borne illness. Additional infectious workup included Babesia, Bartonella, Coxiella, Brucella, Legionella, cytomegalovirus, and fungal serology, all of which returned negative (Table 1).

**Table 1:** Infectious disease workup

Workup	Lab Value	Reference Range
SARS-CoV-2 rRT-PCR	Negative	Negative
Influenza A/B NAAT	Negative	Negative
Anaplasma/Ehrlichia blood smear	None Seen	None Seen
Anaplasma/Ehrlichia PCR	Negative	Negative
Lyme disease	Nonreactive	Nonreactive
Babesia microti IgG and IgM	<1:64 IgG and <1:20 IgM	<1:64 IgG and <1:20 IgM
Bartonella henselae IgG and IgM	<1:128 IgG and <1:20 IgM	<1:128 IgG and <1:20 IgM
Coxiella (Q fever) IgG and IgM	<1:16 IgG and IgM	<1:16 IgG and IgM
Brucella abortus IgG & IgM	Negative	Negative
Legionella antigen urine	Presumptive Negative	Presumptive negative
Cytomegalovirus IgG and IgM	Negative	Negative
Aspergillus antibody	Negative	Negative
Blastomyces antibody	Negative	Negative
Coccidioides antibody	Negative	Negative
Histoplasma antibody	Negative	Negative
West Nile virus IgM	Positive	Negative

On day 4 of hospitalization, the patient reported increased shortness of breath and central chest discomfort. Chest X-ray did not show any focal consolidation, pleural effusion, or pneumothorax. Troponin was mildly elevated, peaking at 0.048 (reference range 0.000-0.033 ng/mL), and B-type natriuretic peptide was 333 (reference range 0-100 pg/mL). The electrocardiogram did not show acute ischemic changes; however, a transthoracic echocardiogram (TTE) showed new global left ventricular hypokinesis compared to one performed 2 months prior (Video 1).

**Video 1:** Transthoracic echocardiogram showing new global left ventricular hypokinesis and ejection fraction of 40%

His ejection fraction also decreased from 55% to 40%. The patient had clinical signs of volume overload including shortness of breath, elevated brain natriuretic peptide (BNP), basilar crackles, and bilateral lower extremity edema, which in combination with his echocardiogram findings was consistent with acute decompensated heart failure with reduced ejection fraction. The patient’s shortness of breath was resolved with diuretic therapy.

The patient’s fevers resolved over the course of his hospital stay, and eventually, results for West Nile IgM antibodies returned positive. After marked clinical improvement, a repeat echocardiogram was performed a week later. It showed a return of the ejection fraction back to his baseline of 55%. No pericardial effusion was noted, and regional wall motion abnormalities had also improved; therefore, additional testing for myocarditis, including myocardial biopsy and cardiac magnetic resonance imaging (MRI), was not performed (Video 2).

**Video 2:** Transthoracic echocardiogram showing return of ejection fraction back to baseline of 55% and improved regional wall abnormalities

Of note, his neurological exam was normal throughout the entire hospitalization, and headache and dizziness symptoms resolved, making significant WNV neuroinvasive disease unlikely. The patient improved throughout his hospitalization and was discharged to home with home health.

## Discussion

Based on the troponin elevation, decreased ejection fraction with subsequent improvement, and positive WNV IgM antibodies, the patient’s presentation was suggestive of West Nile virus-associated cardiomyopathy. Currently, very few cases of West Nile virus-associated myocarditis or cardiomyopathy have been reported. WNV infection is diagnosed via the detection of IgM antibodies in serum or CSF specimens using an enzyme-linked immunosorbent assay (ELISA) test with sensitivity and specificity both greater than 95% [4]. Diagnosing viral myocarditis is more difficult, as the only definitive test is through post-mortem examination with findings of myocardial necrosis and lymphohistiocytic myocarditis. Given the improvement in his ejection fraction and wall motion abnormalities, additional testing such as cardiac MRI was not pursued in this case. Therefore, we are unable to determine if the patient had definite myocarditis. One prior case reported by Kushawaha et al outlined a case of neuroinvasive disease associated with myocarditis. Cardiac pathology in that case was consistent with myocarditis, showing left ventricular scarring as well as lymphocytic and histiocytic inflammatory infiltrates [5]. Whether an indirect immune response to the WNV infection or direct viral infiltration of cardiac myocytes and associated inflammation is the primary pathology in WNV-associated myocarditis, remains to be determined.

Additional cases described have shown evidence of cardiomyopathy similar to our patient without definite findings of myocarditis. Pergam et al. described a 69-year-old man presenting with diffuse weakness, fever, headache, neck stiffness, diarrhea, and nausea. The patient had progressively worsening troponin and an eventual decrease in ejection fraction at 45-50%. WNV serology eventually resulted positive for IgM antibodies. Unfortunately, the patient deteriorated further, and support was withdrawn. A post-mortem examination was declined; however, the authors felt the atrial flutter/fibrillation, new global hypokinesis, reduced ejection fraction, and elevated troponin to be consistent with diffuse myocardial damage secondary to WNV-associated myocarditis [6]. Another case published by Khouzam was associated with more severe cardiomyopathy. The author described a 42-year-old woman with diffuse weakness, body aches, fatigue, low-grade fever, and dyspnea upon mild exertion for a few weeks. Serum WNV antibodies were positive, and her presentation, along with mild cardiomegaly incidentally noted on the computed tomography (CT), prompted further investigation. TTE showed global hypokinesis without regional wall motion abnormalities and an ejection fraction of 25-30% with negative cardiac enzymes and decreased BNP [7]. Compared to the cases outlined above, the current case was relatively mild, with a quick reversal of symptoms. However, clinicians should still be aware of the uncommon complications of WNV infection, including myocarditis or cardiomyopathy for prompt recognition and management.

Aside from the unusual manifestation of cardiomyopathy, the patient in our case generally had symptoms classically associated with WNV. The incidence of WNV infection in the United States is highest in the Midwest during the summer months of July to September, which was when and where our patient presented [3]. His recent travel to Alaska was likely a confounder in this case given WNV is uncommon in Alaska. While 80% of infected individuals are asymptomatic, patients who are symptomatic primarily experience a mild flu-like illness, termed WNF, after an incubation period of 2-14 days, similar to our patient in this case. Low-grade fever, headache, myalgias, nausea, vomiting, and fatigue are common symptoms, although a transient rash may also appear and usually lasts less than a day [2,3]. A very small subset, about 1%, of symptomatic individuals progress to a neuroinvasive disease, which may be meningitis, encephalitis, or acute flaccid paralysis from anterior myelitis [1-3]. Though our patient did have some findings suggestive of possible neurologic manifestations with headaches and dizziness, he did not have significant neurologic involvement. Those with underlying risk factors such as older age, hematological malignancies, and immunosuppression are at greater risk for neuroinvasive disease. Other WNV-associated complications include rhabdomyolysis, hepatitis, and pancreatitis, which were not present in this case [1].

To date, there are no human vaccines or effective antivirals to prevent or treat WNV infection. The only WNV vaccines licensed for use in the United States are for horses [1,2]. Pharmacological agents such as the antiviral ribavirin, interferon alpha-2b, and WNV-specific neutralizing monoclonal antibodies, among others, have been previously studied. Directed therapies would be particularly helpful for patients such as our current patient given his immunosuppressed state, but studies have been limited by an insufficient number of patients or have only shown efficacy in animal models but not in humans [2,3]. The current standard of care is the supportive treatment of fluid balance, antiemetics for nausea and vomiting, in addition to analgesics for myalgias and arthralgias. However, it is still important to promptly recognize the complications of WNV infection, such as arrhythmias or signs of volume overload that can point toward myocarditis or congestive heart failure, as this will lead to overall better management of these patients.

Importantly, preventative measures such as community mosquito control programs and personal protective gear are the primary methods of prevention against WNV infection [1,2]. Limiting outdoor activities during high mosquito activity times of dawn and dusk, wearing long sleeves and pants, and using insect repellents are individual approaches to prevention. Community control programs include targeting breeding sites and spraying for adult mosquitoes [1]. As local and regional outbreaks are unpredictable, clinicians should consider WNV infections, especially during summer months and when the history suggests exposure to mosquitoes through outdoor activities, when approaching patients with complaints of fever, headache, myalgia, and fatigue.

## Conclusions

The current case represents a rare example of WNV-associated cardiomyopathy. Testing for WNV in endemic areas with findings classic of viral illness would be suggested. Additionally, it is important to be mindful of WNV-associated complications and act quickly to appropriately discover potential cardiac pathology through testing such as echocardiography. Rapid treatment of complications, such as arrhythmias, will lead to better management and care of these patients.
